# Learning Disabilities Elevate Children’s Risk for Behavioral-Emotional Problems: Differences Between LD Types, Genders, and Contexts

**DOI:** 10.1177/00222194211056297

**Published:** 2021-11-13

**Authors:** Tuija Aro, Kenneth Eklund, Anna-Kaija Eloranta, Timo Ahonen, Leslie Rescorla

**Affiliations:** 1 University of Jyväskylä, Finland; 2 Niilo Mäki Institute, Jyväskylä, Finland; 3 Bryn Mawr College, PA, USA

**Keywords:** learning disabilities, reading disability, math disability, behavioral-emotional problems, ASEBA

## Abstract

Our purpose was to study the frequency of behavioral-emotional problems among children identified with a learning disability (LD). The data were obtained for 579 Finnish children (8–15 years) with reading disability (RD-only), math disability (MD-only), or both (RDMD) assessed at a specialized clinic between 1985 and 2017. We analyzed percentages of children with behavioral-emotional symptoms reaching clinical range (i.e., *z* score ≥1.5 *SD*s) and the effects of the LD type, gender, and context (home vs. school) on them. Furthermore, we analyzed the effect of the severity of LD and gender on the amount of behavioral-emotional symptoms reported by teachers and parents. Alarmingly high percentages of children, irrespective of LD type, demonstrated behavioral-emotional problems: more than 37% in Affective, Anxiety, and Attention-Deficit/Hyperactivity Disorder (ADHD) problems. Contextual variation was large, as more problems were reported by teachers than by mothers. The unique effects of gender and LD type were rare, but the results raised concern for those with MD-only, especially boys. The results underscore the need to draw attention to the importance of assessing children with LD for behavioral-emotional problems and emphasize the importance of teachers’ awareness of behavioral-emotional problems among students with LD and cooperation among child, teacher, and parents in assessment and support planning.

The co-occurrence of behavioral-emotional problems and learning disabilities (LDs; i.e., difficulties in learning academic skills in reading and math) has been clearly shown among children (Maag & Reid, 2006; Nelson & Harwood, 2011a, 2011b). Research has also indicated that this co-occurrence has implications for intervention and long-term outcomes, as a negative effect of externalizing problems for intervention gain among children with math disability (MD) has been found (Benz & Powell, 2020), and co-occurring reading difficulties and behavior problems have been found to increase the risk of poorer educational attainment (Smart et al., 2017). Furthermore, longitudinal studies have shown that psychiatric problems in adolescence mediate between childhood LD and adult-age psychiatric problems (Eloranta et al., 2021) and that childhood LD are associated with adverse outcomes even in adult age in education, employment, and psychological well-being (e.g., T. Aro et al., 2019; Eloranta et al., 2019; Maughan & Carroll, 2006; McLaughlin et al., 2014). Furthermore, childhood behavioral-emotional problems alone have been linked with failures in achieving social and educational milestones (National Research Council and Institute of Medicine [NRC and IoM], 2009; Reid et al., 2004). These findings indicate that behavioral-emotional problems and LD interact and may lead to less favorable intervention effects and adult-age outcomes. However, we still lack knowledge on the association between different types of LD and behavioral-emotional problems and on how consistently boys and girls display behavioral-emotional concerns across different contexts, that is, at home and at school (cf. contextual variation). Better understanding of specific associations could improve recognition of subclinical problems and identification of children most in need of support for both emotional well-being and academic skills, as well as guide prevention and intervention development in considering behavioral-emotional problems among children with LD.

Behavioral-emotional problems are commonly defined using two dimensions: *externalizing* and *internalizing* problems (e.g., Achenbach & Rescorla, 2001). The first refers to aggressive, oppositional defiant, and hyperactive-impulsive behavior and conduct disorder, and the second to withdrawal, depression, somatization, and anxiety. Higher levels of both types of symptoms have been reported among children with LD (e.g., Nelson & Harwood, 2011a, 2011b). However, in previous LD research, the focus has often been on internalizing or externalizing behavior as broad concepts without specification of separate scales, or on one specific syndrome (e.g., depression). Most of the studies have included only one type of LD (mostly reading disability [RD]) or specification of the LD has not been made. Only a few studies have studied children with MDs or comorbid reading and math disability (RDMD), although the high rate of comorbidity is known (Joyner & Wagner, 2020) and there are indications that problems in learning math increase the risk for emotional problems (T. Aro et al., 2019; Parhiala et al., 2018; Sorvo et al., 2017). Because previous research on behavioral-emotional problems has frequently overlooked problems faced by students with MD or RDMD in different contexts, further research comparing different types of LD and including different internalizing and externalizing symptoms occurring both in school and/or at home is needed. Besides, the effect of the severity of academic difficulty has not been studied, and gender differences have not been consistently examined.

In previous studies, identification of individuals with LD has varied in several aspects, making it complicated to draw conclusions. First, the performance criterion for identification has varied: For instance, performance 1.25 (e.g., Willcutt et al., 2013) or 2.0 *SD*s (Heiervang et al., 2001) below age- or grade-level, or belonging to the lowest 5th (e.g., Auerbach et al., 2008), 10th (e.g., Graefen et al., 2015), or 18th (e.g., Arnold et al., 2005) percentile, have been used. Accordingly, with the lack of clear consensus, different terms (e.g., disability, difficulty, and poor performance) have been used, sometimes interchangeably. Second, population-based samples (e.g., Heiervang et al., 2001) and samples previously identified as having LD (e.g., Carroll & Iles, 2006) have been used. Third, identification has been based either on a test battery (e.g., Auerbach et al., 2008) or on a single test (e.g., Arnold et al., 2005), and fourth, discrepancies between academic performance and IQ have been used in some studies (e.g., Martínez & Semrud-Clikeman, 2004; Miller et al., 2005). All the approaches designating participants categorically to those with and without LD have to use a pre-set cutoff score, which is always arbitrary to some extent. In the present study, we used clinical data of children carefully assessed with a comprehensive neuropsychological test battery, and all participants performed below or at −1.5 *SD*s in a normed test of reading, math, or both compared with grade expectations. We thus used the term *learning disability*.

With the lack of clear consensus, and the different criteria and methods, no conclusion can be made concerning the effect of the LD’s severity on behavioral-emotional problems. For instance, Miller et al. (2005) found that children at the lowest end of the reading distribution were not more likely to have significant internalizing symptoms than children with less impaired reading, but contrary results emerged in math, as Wu et al. (2014) found an association with math achievement and externalizing, but not with internalizing symptoms. However, math anxiety differed between children classified as MD (<10th percentile), low achieving (11th–24th percentile), or typical (>40th percentile), and the authors conclude that even in nonclinical samples, math difficulties are associated with attentional difficulties and math anxiety. Moreover, somewhat contradictory findings have been reported even with the same criterion: Using the 18th percentile as the cutoff, Arnold et al. (2005) found no differences between those performing below the cutoff and typically developing peers, whereas Goldston et al. (2007), using the same criterion, found differences. These findings raise the concern that if scientific studies or individual assessments are conducted only categorically based on specific criteria, there is a risk that the well-being problems of those with less severe academic problems are overlooked and poorly understood.

Using both categorical and continuous approaches, we were able to analyze not only different behavioral-emotional problems demonstrated in different contexts (i.e., reported by teacher or by parent) among boys and girls with RD-only, MD-only, or RDMD, but also the effect of the severity of the academic difficulty. As all these factors (type of LD, gender, context, severity of LD) are relevant, they need to be considered when aiming to understand the individual child and planning support. In addition, more research considering different factors in concert is needed to better understand how they should be incorporated into our future theoretical models of developmental psychopathology.

## Effects of Type of Learning Disability

The studies targeting RD identified based on performance being at least below 10th percentile have shown its association with internalizing symptoms such as anxiety, depression, somatic complaints, and withdrawal (Carroll et al., 2005; Livingston et al., 2018; Mammarella et al., 2016; Willcutt & Pennington, 2000). However, in some studies, differences in self-reported depression between children with RD and controls were not detected (Carroll et al., 2005; Heiervang et al., 2001; Miller et al., 2005). Also, externalizing symptoms such as aggressive (Willcutt & Pennington, 2000) and delinquent behavior (Willcutt & Pennington, 2000) have been reported, but the majority of the studies have reported internalizing problems without considering the context (i.e., home or school).

Although much less studied, in studies identifying MD based on performance below at least the 10th percentile, MD has been associated with internalizing problems such as math anxiety (e.g., Wu et al., 2014), generalized anxiety, and major depressive disorder (e.g., Willcutt et al., 2013), eating disorders, somatization, and hypochondria (Graefen et al., 2015). The recent findings by T. Aro et al. (2019) among adults indicated that MD identified in childhood was associated with high antidepressant use during the adults’ life courses. Some studies have also reported externalizing problems such as Oppositional Defiant Disorder and Conduct problems (Auerbach et al., 2008; Willcutt et al., 2013), but again the focus has mostly been on internalizing problems and the context has not been analyzed.

There are even fewer studies on RDMD than on RD-only or MD-only. The few studies comparing problems among individuals with RDMD and those with a single deficit have produced contradictory results. Willcutt et al. (2013) found that the RDMD group showed more internalizing problems (generalized anxiety, major depressive disorder) than the groups with single deficits, whereas Martínez and Semrud-Clikeman (2004) did not find differences among RD, MD, and RDMD. To fill the gap in knowledge concerning MD and RDMD and to shed more light on contradictory findings on RDMD, we analyzed behavioral-emotional symptoms reported by parents and teachers among children diagnosed with RD and/or MD.

There are some differences between basic reading and math as school subjects, which may affect the psychological well-being of the child facing difficulties in them. Difficulties in gaining grade-level fluent reading skills are easily observed by children, as oral reading is used in early reading instruction, which provides a visible point of comparison to peers. This would make a child with RD vulnerable to negative self-concept. Later on, dysfluent reading (which is the focus of the present study) may cause difficulties in reading comprehension, and hence burden students in other subjects. On the contrary, math includes distinct areas to learn (e.g., number facts, arithmetic, algebra, geometry), and different math skills are based on different cognitive processes (McCloskey & Caramazza, 2018). Math is also a cumulative subject (the learning of new content is based on the mastering of earlier content), and therefore, a child with MD may be faced over and over again with his or her difficulties. Math disability has also been shown to be associated with math-related anxiety (e.g., Carey et al., 2017), and strong emotions and negative meanings may emerge in association with math (Lange & Meaney, 2011; Takeuchi & Martin, 2018). Furthermore, although there are several shared cognitive deficits associated with RD and MD (e.g., Willcutt et al., 2013), deficits in executive functions have been found to be related especially to MD (Cragg et al., 2017; Willcutt et al., 2013; see however about executive functions and reading development and reading comprehension: Cirino et al., 2019; Follmer, 2018; Haft et al., 2019). As executive functions are also relevant for emotion regulation (e.g., Hendricks & Buchanan, 2016; Zelazo & Cunningham, 2007), these deficits may predispose especially children with MD to behavioral-emotional problems. Thus, differences between LD subtypes in behavioral-emotional problems may stem from the differences in reading and math as school subjects, emotions attached to these subjects, and cognitive deficits related to RD and MD. However, it is not possible to draw specific hypotheses about differences in behavioral-emotional problems related to RD and MD either at home or at school.

## Effects of Gender

Population-based studies have shown that girls are more prone to somatic disorders, depression, and anxiety, whereas boys are more prone to oppositional defiant disorder, conduct disorder, and attention-deficit/hyperactivity disorder (ADHD; Altemus et al., 2014; Martel, 2013). The findings on gender differences in behavioral-emotional problems associated with LD are not as consistent. Some studies have suggested higher levels of depressive symptoms among girls (Heath & Ross, 2000; Martínez & Semrud-Clikeman, 2004), but many studies have not reported their results by gender or have not found gender differences (Maag & Reid, 2006; Nelson & Harwood, 2011a, 2011b), and only a few have analyzed different types of LD in concert.

Research on gender effects among students with LD has found somewhat contradictory results. Studies focusing on RD suggest that girls are more likely to experience internalizing problems, such as depression or anxiety, compared with boys with RD (Nelson & Gregg, 2012; Willcutt & Pennington, 2000), who have been found to have more externalizing problems than girls and controls (Heiervang et al., 2001; Willcutt & Pennington, 2000). However, Carroll et al. (2005) found that more teen-age boys with RD self-reported depression than did girls. The findings concerning MD are similarly confusing, however, suggesting that there might exist gender-related differences. Wu et al. (2014) found that the relation between math achievement (among MD, low achieving, and typical) and externalizing problems was stronger among girls than boys, and Graefen et al. (2015) reported higher ratings on internalizing problem scales among boys than girls. Conclusions on the interaction between gender and LD type cannot be drawn, and further research analyzing gender effects in behavioral-emotional problems among different LD types is needed.

## Home Versus School Contexts

LD manifest mainly in the school context, and it is not surprising that children with LD tend to compare their performance with that of their peers, have negative self-concept (e.g., Gans et al., 2003) and lower self-efficacy (Hampton & Mason, 2003; Peura et al., 2019), and have difficulties integrating socially (Gadeyne et al., 2004). Because LD may affect the construction of self (e.g., Humphrey & Mullins, 2002), behavioral-emotional problems are likely not to be restricted to school. However, the information gained from parents and teachers often differs (cf. informant discrepancy or low cross-informant agreement; De los Reyes & Kazdin, 2005; van der Ende et al., 2012), which causes uncertainty in decision-making (de los Reyes et al., 2013). Especially low agreement between parent and teacher reports has been found concerning internalizing problems in both community (Youngstrom et al., 2000) and clinical samples (Salbach-Andrae et al., 2009; Stanger & Lewis, 1993).

There is a paucity of studies scrutinizing informant discrepancy in association with LD, and thereby, we lack knowledge on the problems occurring among children with LD at home (parent as the informant), in school (teacher as the informant), or in both contexts. Most previous LD studies have utilized reports of solely parent (Auerbach et al., 2008; Willcutt & Pennington, 2000) or solely youth self-reports (e.g., Mammarella et al., 2016). The mean of parents’ and teachers’ ratings has also been used, but reports were not compared (Carroll et al., 2005; Willcutt et al., 2013). Nelson and Harwood (2011a) did not find differences between results based on parent reports and those based on teacher reports in meta-analysis on LD and depression, but Dahle et al. (2011) found that parents reported more children with RD to be anxious and depressed and to have more attention problems than did teachers.

The interpretation of informer discrepancy is not straightforward. Rather than interpreting it as an indication of measurement error, it could be understood as an indication of contextual and interactional differences in the manifestation of the problems or different perceptions of the informants. The underlying assumption of multi-informant assessment is the situational specificity of the problems. Home and school have different structures, interactional relationships, and sources of support, and the function of the child’s behavior may differ according to the context. For example, learning situations possibly leading to failure and frustration (maybe later to aggression; see Miles & Stipek, 2006) or embarrassment (maybe later to negative self-related emotions; see Chapman et al., 2000) may cause an urge to avoid instructional activities at school, while the same experience may evoke attention or consolation seeking behavior at home, as home might provide a safer context for expressing distressing emotions. Although the reasons for low cross-informant agreement are beyond the scope of this study, the earlier findings underscore the importance of understanding informant variance and considering several informants among children with LD. A better understanding of the similarities and differences in parent and teacher perceptions in different types of LD may inform us about the cross-situational generality of behavioral-emotional problems and indicate the pervasiveness of symptoms.

## Categorical Versus Dimensional Assessment of Behavioral-Emotional Problems

Several previous studies focused on *mean* level differences between children with and without LD on ratings of psychological well-being, and their findings do not necessarily indicate whether participants had higher rates of *clinical* disorders (Maag & Reid, 2006), as the symptom levels could have been within the normal range for all groups. Therefore, we lack knowledge on the amount of behavioral-emotional problems reaching clinical range among children with LD, and especially on whether they manifest to the same extent in different contexts. Thus, we adopted two approaches to study these problems and used instruments that allowed us to score using both categorical (above or in clinical range, i.e., *z* score ≥ 1.5 *SD*s compared with the normative sample) and quantitative scales (dimensional), that is, Child Behavior Checklist (CBCL) and Teacher Rating Forms (TRF; Achenbach & Rescorla, 2001). We first analyzed whether different LD groups manifested different percentages of children scoring in the clinical range, according to teacher, mother, or both. Although we did not aim to diagnose the participants of the study, using the *DSM*-oriented scales from the CBCL/TRF allowed for partition of the problems according to the prevailing nosology, as the scales have been found to be consistent with the diagnostic categories of the *Diagnostic and Statistical Manual of Mental Disorders–Fourth Edition* (*DSM-IV*; American Psychiatric Association, 1994; Achenbach & Rescorla, 2001). Second, we studied the degree to which problems were reported in different contexts, and third, whether the severity of the learning difficulties affected the amount of behavioral-emotional symptoms. As even subclinical symptoms may cause considerable concern for the child, targeting only problems in the clinical range would not provide the overall sense of the association between LD and behavioral-emotional symptoms.

## Goals of the Study

The first aim was to determine the percentages of boys and girls with RD-only, MD-only, and RDMD scoring in the clinical range (i.e., *z* score ≥1.5 *SD*) on the six *DSM*-oriented scales of the CBCL or TRF (categorical approach). We then examined how context (home vs. school) affected these percentages. The second goal was to test the effects of RD and MD severity and gender on quantitative scores of the *DSM*-oriented scales (dimensional approach). The research questions were as follows:

**Research Question 1 (RQ1):** What percentage of boys and girls in the three LD groups showed problems in the clinical range?
**Research Question 2 (RQ2):** What percentage of children showed them solely at home, solely in school, or in both contexts?
**Research Question 3 (RQ3):** To what extent did severity of reading and math difficulty and gender explain CBCL and TRF scale scores?

## Method

### Procedure and Participants

The sample was derived from the archival client database of the Clinic for Learning Disorders (CLD), which is a public clinic affiliated with the Niilo Mäki Institute (NMI) and Jyväskylä City’s Family Counseling Center. It provides free services for families in Central Finland. Parents have given informed consent to use the data for research purposes, and the institutional consent to use the data was provided by the Ethics Committee of the University of Jyväskylä. The CLD has offered assessment and counseling for children with LD (typically 7–13 years of age)—mainly referred by the Family Counseling Center or school psychologists—since 1985. There are no formal exclusionary criteria, but children with behavioral-emotional symptoms as their primary problems are not referred to the CLD, and only children with noticeable and prolonged difficulties in academic performance are referred. Before referral, the difficulties will have first been noticed by classroom teachers (or parents) and assessed by special education teachers, and individually planned and/or intensified educational support been provided. It should be noted that a special education teacher with master’s degree is available in every school, and each class has an appointed special education teacher working in close collaboration with the classroom teacher. No formal diagnosis is needed for special educational support. If the problems persist despite the intensified support, the school psychologist or a decision-making team consisting of administrators, teachers, school psychologists, and the parents is involved in the assessment process and support planning (see Björn et al., 2016). If these measures turn out to be insufficient, the child is referred to the CLD. Thus, the process closely resembles the Response to Intervention model used in the United States (e.g., Fletcher & Vaughn, 2009). This multitiered framework with systematized assessment and instruction, cyclic support, and modifiable instruction has already been used in the 1980s and has been officially implemented in Finland since 2010.

At the CLD, a comprehensive assessment that includes neuropsychological testing, reading and math testing, and parental and teacher ratings of behavioral-emotional symptoms is conducted. The tests used have varied over the years, and clinical judgment has been used in choosing relevant measures. As a result, several measures were used when assessing children in the present sample, and some children had missing data for some measures. Individuals with age and/or grade, gender, and both reading and math scores available were included if they clearly demonstrated LD; that is, their performance was at least 1.5 *SD*s below the mean of the reference group in reading and/or math tests conducted during the individual assessment at the CLD. The cutoff of 1.5 *SD* was chosen as it corresponds to 7th percentile in normal distribution and concurs with several previous studies on LD. There were 1,234 children’s data in the database, and only those children with both reading and math scores available were included. There were 1,001 children who had a reading test score, 932 who had a math test score, and 830 who had both scores available; of them, 632 had either score ≤−1.5 *SD.* Thus, children identified as having RD-only had math scores above −1.5 *SD*, and children with MD-only had reading fluency scores above −1.5 *SD*, and in the case where both scores were ≤−1.5 *SD*, the child was identified as having RDMD. The IQ score was not used when defining LD, but we excluded children with IQs < 75. Of the 632 children, 14 children with IQ scores below 75, 22 with missing IQ scores, and 17 with missing CBCL and TRF scores were excluded. This procedure yielded a final sample of 579 children: 368 (63.6%) boys and 211 (36.4%) girls. The mean age was 10.31 years (*SD* = 1.18 years; grade *Mdn* = 4; IQ *M* = 89.55, *SD* = 10.61). The data were saved digitally until 2017, and the participants were assessed as follows: 91 (15.6%) during 1985–1994, 218 (37.5%) during 1995–2004, 237 (40.7%) during 2005–2014, and 33 (5.7%) during 2015–2017. When analyzing percentages of children reported to have behavioral-emotional problems in these four cohorts, we noticed that mothers reported more Anxiety problems and teachers reported more Affective, Anxiety, and ADHD problems in the first cohort assessed between 1985 and 1994. Therefore, we also analyzed the data without the first cohort, and the results reported in the Results section were corroborated with this smaller data except that the difference between girls and boys in the RD-only group concerning Affective problems was no more significant.

### Measures

#### Measures of reading fluency

Reading disability was defined on the basis of *reading fluency* because in orthographically transparent languages, like Finnish, children achieve accurate reading skills mostly during the first grade, after which RD is manifested mainly as dysfluency (M. Aro, 2004). In this study, reading fluency refers to reading rate and accuracy, whereas prosody is not considered. The definition of RD definition was based on child’s reading fluency in one of the following text or wordlist reading tests commonly used by psychologists to assess reading skills. They have norms collected locally, but unfortunately psychometric information is not available except for the Lukilasse. The Misku-Text (Niilo Mäki Institute [NMI], 1985–2004) is a text-reading task normed for 8- to 12-year-old children, in which the child is to read aloud a short story as fluently and correctly as possible. The Ärps (NMI, 1985–2004) is a word- and pseudo-word reading test normed for Grades 2 to 4 (five children in this study were identified based on their fluency in Ärps pseudo-word reading). The Markkinat Word List (NMI, 1985–2004), also normed for 8- to 12-year-old children, consists of 13 words that the child is to read aloud as fluently and accurately as possible. The Lukilasse (Häyrinen et al., 1999) is a reading, spelling, and math skills test battery normed for Grades 1 to 6. In the Word Reading subtest, the child reads aloud a list of words that gradually become longer and more difficult. The fluency score is obtained by calculating the correctly read words within 2 min. Cronbach’s alphas ranged between .94 and .98, depending on the grade (Häyrinen et al., 1999).

#### Measures of math skills

MD definition was based on one of the following tests. The Kaufman Assessment Battery for Children–Arithmetic Subtest (K-ABC; Kaufman & Kaufman, 1983) includes 38 tasks measuring children’s knowledge of numbers, mathematical concepts, and computational skills. The internal consistency values of the K-ABC subtests have been found to be at least .86 among school-age children. Local norms are available for Grades 2 to 5 (NMI, 1985–2004). In the RMAT (Räsänen, 1992; normed for Grades 3–6), the child is requested to perform as many basic arithmetical operations (max. 55) as possible in 10 min. The test has been shown to have high internal validity and reliability with Cronbach’s alpha of .86 and test–retest reliability (*r* = .82, 6 months interval and *r* = .76, 14 months). The Lukilasse Arithmetics Subtest (Häyrinen et al., 1999) consists of basic arithmetic operation tasks normed for Grades 1 to 6. Cronbach’s alpha of the test ranged between .55 and .83, depending on the grade (Häyrinen et al., 1999).

#### Measures of behavioral-emotional problems

Behavioral-emotional problems were rated by parents using the CBCL and by teachers using TRF from the Achenbach System of Empirically Based Assessment (ASEBA; Achenbach & Rescorla, 2001). CBCL/6-18 parent forms were completed by either the mother or father (or surrogates), and TRF forms were completed by teachers. From the parental reports, the form filled out by the mother was used, as fewer father reports were available. In the case of a missing mother’s report, the father’s report was used. The battery has been used in numerous societies to assess behavioral and emotional problems (Rescorla et al., 2007) and has good cross-cultural consistency (Crijnen et al., 1997). We used the six *DSM*-oriented scales developed by international expert panels who identified items that they judged to be very consistent with particular *DSM-IV* diagnostic categories (Achenbach & Rescorla, 2001). Similar factor structures, internal consistency, and mean scores in samples collected in several societies have been found (Ivanova et al., 2007; Rescorla et al., 2007, 2012). Cronbach’s alphas of the *DSM*-oriented scales have been reported to range from .75 to .84 (*M* = .80) and the mean test–retest *r*s to range from .78 to .88 (*M* = .83) (Achenbach et al., 2003). The alpha coefficients for the six *DSM*-oriented scales of the CBCL scales have varied from .58 to .75 across the 31 societies (the lowest alphas were found in Anxiety problems and Somatic problems scales; Rescorla et al., 2007). All CBCL and TRF *DSM*-oriented scales have been shown to have high validity for both clinical and nonclinical populations and to differentiate the samples, as all scale scores were significantly lower for non-referred than referred children (Achenbach & Rescorla, 2001).

Two population-based Finnish normative samples (Rescorla et al., 2007), one with parental ratings (CBCL) and one with teacher ratings (TRF), were used to calculate standardized scores for the scales in the current sample. The CBCL normative sample consisted of 2,093 children (1,021 boys and 1,072 girls; ages 6–15 years). The TRF sample consisted of 1,695 children (834 boys and 861 girls; ages 6–16 years). Both data were based on a regional school-based sample; the parents completed CBCL at school or the child conveyed it to them. The response rate was 77%, and the referred children were not excluded (Rescorla et al., 2007). As the clinical data used in this study have been gathered since 1985, the versions of the questionnaires have changed over the years. Therefore, the few items that were different in the questionnaire versions were excluded, and the scales were calculated similarly for both the clinical data and the normative population-based samples. Of the internalizing scales, 12 items comprised Affective problems in the CBCL and nine in the TRF (e.g., Cries a lot; Feels worthless or inferior), six items comprised Anxiety problems in both CBCL and TRF (e.g., fears certain animals, situations; nervous, tense), and seven items comprised Somatic problems in both CBCL and TRF (e.g., aches, pain; nausea). Of the externalizing scales, seven items comprised Attention-Deficit/Hyperactivity problems in CBCL and 13 items comprised the same scale in the TRF (e.g., can’t concentrate, pay attention; impulsive or acts without thinking), five items comprised Oppositional Defiant problems in both the CBCL and the TRF (e.g., argues a lot; disobedient at home/at school), and 16 items in CBCL and 12 in the TRF comprised Conduct problems (e.g., destroys property belonging to others; mean, cruel to others). The Cronbach’s alphas of the *DSM*-related scales in the CBCL varied between .67 and .82 in our clinical data and between .65 and .78 in the normative data, except in Somatization symptom, where it was between .58 and .52 (clinical and normative data, respectively). In TRF, the Cronbach’s alphas varied between .64 and .93 and .63 and .94 in the clinical and normative data, respectively.

A cutoff score of 1.5 *SD*s (similar to identifying children with LD) was used as a criterion for manifestation of clinical range problems. This corresponds well to the commonly used cutoff *T* score ≥ 65 in the ASEBA syndrome scales. However, a *T* score ≥69 is suggested for *DSM*-oriented scales (Achenbach & Rescorla, 2001). We used the cutoff 1.5 *SD*s because the referred children were not excluded from the Finnish normative sample. Despite defining our participants as scoring in the clinical range, it should be noted that we do not claim them to have diagnoses of behavioral-emotional problems. A high *DSM*-oriented scale score is not equivalent to a *DSM* diagnosis, as the items of the scales do not correspond precisely to *DSM* criteria: They are quantitative (0–2; whereas in the *DSM* dichotomy is used) and are normed separately for parents and teachers (in the *DSM*, the criteria are the same regardless of the informant; Achenbach et al., 2003; Rescorla, 2005).

#### Measure of intelligence (IQ)

We measured IQ with the Wechsler Intelligence Scale for Children (WISC), of which three versions were used during the time the data for the present study were gathered. Verbal IQ and Performance IQ scores from the Finnish versions of the WISC-R (Wechsler, 1974) and WISC-III (Wechsler, 1991) and the Verbal Comprehension Index (VCI) and the Perceptual Reasoning Index (PRI) from the WISC-IV (Wechsler, 2003) were used. The IQ scores were not used when defining RD or MD.

### Data Analyses

The distribution of reading fluency was left-skewed, whereas mathematical skill was normally distributed. All scale scores of both the CBCL and the TRF were right-skewed, suggesting that a large portion of the children in the sample showed none or only a few behavioral-emotional symptoms. To fulfill the presumption of univariate analysis of variance (multinomial normal distribution), Box-Cox transformations (Osborne, 2010) were performed on all measures with skewed distribution before the analyses. After these transformations, all distributions, except Somatic problems, were normal or close to normal and included no outliers.

Chi-squares were used to analyze the percentages of children scoring in the clinical range on either the CBCL or the TRF *DSM*-oriented scales based on their LD type and gender. The Friedman Test was used to analyze differences in the manifestation of clinical range scores based on context (home, school, or both contexts), and the Wilcoxon signed-rank test was used for the pairwise comparisons of the contexts. Univariate analysis of variance (ANOVA) was used to test the effects of RD and MD severity, gender, and their interaction terms (Gender × RD severity; Gender × MD severity) separately for each of the CBCL and the TRF scales.

## Results

Demographic information is presented in Table 1. The LD groups were similar in terms of age, grade, and IQ indices. There were no statistically significant differences in the distribution of girls and boys within the LD groups. There were more boys than girls in each group, although the gender balance was closer in the MD-only group than in the other groups. As expected, significant group differences were found in reading fluency, *F*(2, 576) = 299.49, *p* < .001, and in math skill, *F*(2, 576) = 376.88, *p* < .001. Children with RD-only and RDMD scored lower in reading fluency than children with MD-only, whereas children with MD-only and RDMD scored lower in math skill than children with RD-only (all *p*s < .001). All effect sizes in pairwise group comparisons were large (Cohen’s *d* varied between 2.45 and 2.70).

**Table 1.** Demographic Information of the Sample With Means and Standard Deviations for Reading Fluency and Mathematical Skill *Z* Scores.

Measure	LD type							
	RD-only^a^ 65/132		MD-only^b^ 61/81		RDMD^c^ 85/155		Total^d^ 211/368	
	*M*	*SD*	*M*	*SD*	*M*	*SD*	*M*	*SD*
Age (months)	125.46	10.01	123.11	14.04	122.73	12.34	123.75	14.15
Grade	3.73	1.48	3.59	1.17	3.60	1.05	3.64	1.24
Verbal IQ/VCI	91.7	12.15	89.99	12.10	88.64	11.62	90.00	11.97
Performance IQ/PRI	94.64	12.85	87.43	13.87	89.12	14.79	90.60	14.22
Reading fluency	−0.30	0.69	1.24	0.54	−0.49	0.79	0.00	1.00
Mathematical skill	1.05	0.51	−0.52	0.67	−0.54	0.75	0.00	1.00

*Note.* Standardized values, based on the means and standard deviation of the current sample (*N* = 579), of the Box-Cox transformed reading fluency and mathematical skill are reported. LD = learning disability; RD = reading disability; MD = math disability; RDMD = reading disability and math disability; VCI = Verbal Comprehensive Index from the Wechsler Intelligence Scale for Children (4th ed.; WISC-IV); PRI = Perceptual Reading Index from the WISC-IV.

a *n* = 197. ^b^*n* = 142. ^c^*n* = 240. ^d^*N* = 579.

### Children Scoring in Clinical Range

All percentages of children scoring in the clinical range were above what would be expected based on the normative data, namely, 7% based on 1.5 *SD* cutoffs (see Table 2). Especially high percentages were found in Affective, Anxiety, and ADHD scales. No differences between genders were found, except that more girls than boys in the RD-only group had Affective problems, χ^2^(1, *N* = 579) = 3.86, *p* = .049. Effect size was small (Cramer’s V = 0.14). Within girls, no differences were found between the LD groups. Within boys, fewer than expected children with RD-only had Anxiety or ADHD problems, but more than expected with MD-only had Anxiety or ADHD problems, χ^2^(1, *N* = 579) = 16.73, *p* < .001, and χ^2^(1, *N* = 579) = 8.66, *p* = .013, respectively. Effect sizes were small both in Anxiety and ADHD (Cramer’s V = 0.21 and 0.15, respectively). No significant effects of LD type or gender were found in Somatic, Oppositional Defiant, or Conduct problems.

**Table 2.** Percentages of Children Showing Clinical Level Behavioral-Emotional Problems in Different LD Groups.

*DSM*-oriented scale	LD type							
	RD-only^a^		MD-only^b^		RDMD^c^		Total^d^	
	Girl	Boy	Girl	Boy	Girl	Boy	Girl	Boy
Internalizing symptoms								
Affective problems	46.2%	31.8%	55.7%	44.4%	40.0%	43.2%	46.4%	39.4%
95% CI	[33.7, 59.0]	[24.0, 40.5]	[42.4, 68.4]	[33.4, 55.9]	[29.5, 51.2]	[35.3, 51.4]	[39.6, 53.4]	[34.4, 44.6]
Anxiety problems	30.8%	25.8%	41.0%	53.1%	41.2%	33.5%	37.9%	35.1%
95% CI	[19.9, 43.4]	[18.5, 34.1]	[28.6, 54.3]	[41.7, 64.3]	[30.6, 52.4]	[26.2, 41.6]	[31.4, 44.8]	[30.2, 40.2]
Somatic problems	21.5%	15.3%	19.7%	24.7%	21.2%	17.4%	20.9%	18.3%
95% CI	[12.3, 33.5]	[9.6, 22.6]	[10.6. 31.8]	[15.8, 35.5]	[13.1, 31.4]	[11.8, 24.3]	[15.6, 27.0]	[14.4, 22.6]
Externalizing symptoms								
ADHD problems	46.2%	38.6%	57.4%	59.3%	55.3%	45.2%	53.1%	45.9%
95% CI	[33.7, 59.0]	[30.3, 47.5]	[44.0, 70.0]	[47.8, 70.0]	[44.1, 66.1]	[37.2, 53.4]	[46.1, 60.0]	[40.8, 51.2]
OD problems	20.0%	22.0%	29.5%	37.0%	28.2%	29.7%	26.1%	28.5%
95% CI	[11.1, 31.8]	[15.2, 30.0]	[18.5, 42.6]	[26.6, 48.5]	[19.0, 39.0]	[22.6, 37.5]	[20.3, 32.5]	[24.0, 33.4]
Conduct problems	16.9%	18.3%	23.0%	30.9%	17.6%	25.8%	19.0%	24.3%
95% CI	[8.8, 28.3]	[12.1, 26.0]	[13.2, 35.5]	[21.1, 42.1]	[10.2, 27.4]	[19.1, 33.4]	[13.9, 24.9]	[20.0, 29.0]

*Note*. LD = learning disability; *DSM* = *Diagnostic and Statistical Manual of Mental Disorders*; RD = reading disability; MD = math disability; RDMD = reading disability and math disability; CI = confidence interval; ADHD = attention-deficit/hyperactivity disorder; OD = oppositional defiant disorder.

a *n* = 196 to 197. ^b^*n* = 142. ^c^*n* = 240. ^d^*N* = 578–579.

### Comparisons Between Contexts

**Figure 1.**
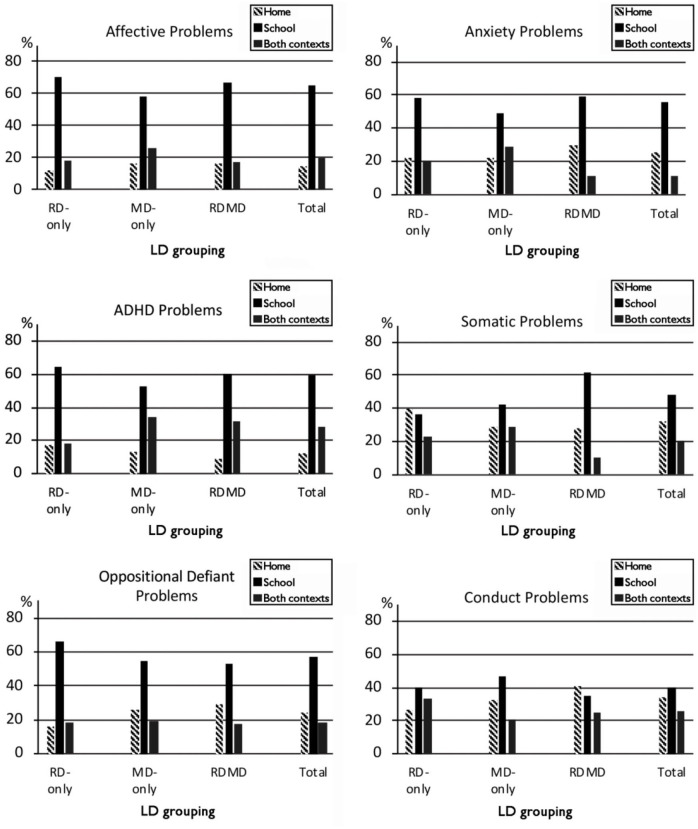
Percentages of Boys and Girls Showing Behavioral-Emotional Problems in Clinical Range Only at Home, Only at School or in Both Contexts.

The Friedman Test showed significant differences between the contexts (home, school, and both contexts) in the manifestation of problems reaching clinical range for all scales except Conduct problems (*N* varies according to the number of children whose symptoms were in the clinical range): Affective, χ^2^(2, *N* = 222) = 103.00, *p* < .001, Anxiety, χ^2^(2, *N* = 190) = 44.25, *p* < .001, ADHD, χ^2^(2, *N* = 252) = 85.45, *p* < .001, Oppositional Defiant, χ^2^(2, *N* = 142) = 36.78, *p* < .001, and Somatic problems, χ^2^(2, *N* = 100) = 11.84, *p* = .003. Pairwise comparisons with Wilcoxon Signed Ranks Test showed that Affective, Anxiety, ADHD, and Oppositional Defiant problems were more often manifested solely in school than at home or both contexts (all *p*s < .001; see Figure 1). Effect sizes were large in Affective (Cohen’s *d* = 1.37 school vs. home; 1.34 school vs. both contexts) and ADHD (1.46; 0.74), and moderate in Anxiety (0.66; 0.85) and Oppositional Defiant problems (0.59; 0.76). ADHD problems were manifested in both contexts more often than solely at home (*p < .*001). Effect size was moderate (0.56). Somatic problems were manifested more often solely in schools than in both contexts (*p < .*001), effect size being moderate (0.47). The difference between solely at home and solely in school was nonsignificant. The relative proportions of problems in different contexts (home, school, and both) were similar in all the LD groups for all scales (see Figure 1).

### Effects of RD and MD Severity on the *DSM-*Oriented Scale Scores

ANOVAs were used separately for each of the CBCL and the TRF scales to examine the effects of RD and MD severity on the scale scores, which were used as continuous dependent measures. Dichotomous gender and continuous Reading Fluency and Math Skill scores indicating the level of difficulty were used as independent measures. The interaction effects Gender × RD Severity and Gender × MD Severity were analyzed. Means and *SDs* for the *DSM*-oriented scale scores by gender are presented in Table 3 separately for the CBCL and the TRF.

**Table 3.** Emotional-Behavioral Problems Based on Parent Ratings on the CBCL and Teacher Ratings on the TRF.

*DSM*-oriented scale	CBCL					TRF				
	Girls^a^		Boys^b^		ES	Girls^c^		Boys^d^		Effect size
	*M*	*SD*	*M*	*SD*		*M*	*SD*	*M*	*SD*	
Internalizing symptoms										
Affective problems	0.43	1.25	0.22	1.09	0.20	1.23	1.85	1.02	1.52	0.12
95% CI	[0.29, 0.63]	[1.09, 1.45]	[0.11, 0.35]	[0.94, 1.24]	[0.03, 0.38]	[0.95, 1.48]	[1.56, 2.12]	[0.87, 1.18]	[1.42, 1.68]	[−0.05, 0.31]
Anxiety problems	0.49	1.31	0.25	1.23	0.19	0.90	1.80	0.80	1.61	0.06
95% CI	[0.31, 0.69]	[1.10, 1.50]	[0.12, 0.39]	[1.15, 1.38]	[0.02, 0.36]	[0.63, 1.12]	[1.49, 2.09]	[0.59, 0.94]	[1.40, 1.83]	[−0.12, 0.24]
Somatic problems	0.27	1.27	0.20	1.11	0.06	0.80	2.36	0.38	1.63	0.22
95% CI	[0.10, 0.46]	[1.07, 1.47]	[0.08, 0.32]	[1.01, 1.22]	[−0.11, 0.23]	[0.46, 1.15]	[1.73, 2.85]	[0.21, 0.55]	[1.25, 1.98]	[0.03, 0.39]
Externalizing symptoms										
ADHD problems	0.60	1.30	0.67	1.21	0.06	1.87	2.18	1.38	1.33	0.29
95% CI	[0.42, 0.78]	[1.11, 1.48]	[0.55, 0.79]	[1.11, 1.30]	[−0.12, 0.23]	[1.58, 2.25]	[1.85, 2.68]	[1.24, 1.52]	[1.23, 1.40]	[0.11, 0.47]
OD problems	0.12	1.14	0.14	1.14	0.02	0.45	1.54	0.64	1.46	0.13
95% CI	[−.03, 0.28]	[1.01, 1.27]	[0.03, 0.28]	[1.04, 1.22]	[−0.15, 0.20]	[0.27, 0.69]	[1.24, 1.81]	[0.50, 0.84]	[1.31, 1.60]	[−0.05, 0.31]
Conduct problems	0.30	1.44	0.30	1.30	0.00	0.31	1.37	0.52	1.39	0.15
95% CI	[0.11, 0.51]	[1.08, 1.78]	[0.17, 0.44]	[1.13, 1.46]	[−0.17, 0.17]	[0.14, 0.53]	[0.95, 1.80]	[0.38, 0.67]	[1.20, 1.55]	[−0.03, 0.33]

*Note.* Effect sizes calculated with Cohen’s *d* using pooled standard deviations of the two groups. *DSM* = *Diagnostic and Statistical Manual of Mental Disorders*; CBCL = Child Behavior Checklist; TRF = Teacher Rating Form, both from the Achenbach System of Empirically Based Assessment (ASEBA); CI = confidence interval; ADHD = attention-deficit/hyperactivity disorder; OD = oppositional defiant disorder.

a *n* = 200. ^b^*n* = 347. ^c^*n* = 189. ^d^*n* = 325.

For the CBCL, we found a significant main effect of MD severity in ADHD, *F*(1, 543) = 7.73, *p* = .006, $\eta_{p}^{2}=.01$ , Oppositional Defiant, *F*(1, 543) = 11.26, *p* < .001, $\eta_{p}^{2}=.02$ , and Conduct problem symptoms, *F*(1, 541) = 5.05, *p* = .025, $\eta_{p}^{2}=.01$ . More severe MD resulted in increased externalizing symptoms. All effect sizes were small. The main effect of RD severity was nonsignificant in all CBCL scales, and the main effect of gender was significant only in Anxiety symptoms, *F*(1, 543) = 8.92, *p* = .003, $\eta_{p}^{2}=.02$ , with girls showing more symptoms. In Anxiety symptoms, there was a significant Gender × MD Severity interaction effect, *F*(1, 543) = 6.14, *p* = .014, $\eta_{p}^{2}=.01$ , and in Somatic symptoms, a significant Gender × RD Severity interaction effect was found, *F*(1, 543) = 6.98, *p* = .008, $\eta_{p}^{2}=.01$ . Further analysis separately by gender revealed that only among boys more severe MD added Anxiety symptoms, *F*(1, 346) = 11.65, *p* < .001, $\eta_{p}^{2}=.03$ , and more severe RD added Somatic symptoms, *F*(1, 346) = 10.96, *p* = .001, $\eta_{p}^{2}=.03$ . Again, both effect sizes were small.

Analyses for the TRF resulted in a significant main effect of MD severity in ADHD, *F*(1, 530) = 24.92, *p* < .001, $\eta_{p}^{2}=.05$ , Affective, *F*(1, 534) = 9.92, *p* = .002, $\eta_{p}^{2}=.02$ , Conduct, *F*(1, 534) = 5.15, *p* = .024, $\eta_{p}^{2}=.01$ , Oppositional Defiant, *F*(1, 540) = 7.59, *p* = .006, $\eta_{p}^{2}=.01$ , and Somatic symptoms, *F*(1, 517) = 9.60, *p* = .002, $\eta_{p}^{2}=.07$ , but not in Anxiety. More severe MD resulted in increased symptoms. All effect sizes were small. The main effect of RD severity was nonsignificant in all TRF-scales and the main effect of gender was significant in Somatic symptoms, *F*(1, 517) = 39.66, *p* < .001, $\eta_{p}^{2}=.02$ , with girls showing more symptoms. Effect size was intermediate.

## Discussion

We studied the associations between LD and behavioral-emotional problems among 579 children (ages 8–15 years) diagnosed as having RD-only, MD-only, or RDMD. The analyses indicated that high percentages of children with LD, irrespective of the LD type, demonstrated behavioral-emotional symptoms in the clinical range (i.e., *z* score ≥1.5 *SD*). A large contextual variation was found, as the problems were manifested most often in the school context, that is, they were reported by teachers. Gender- or LD-type-specific findings were rare, but the results raised special concern for children with MD-only.

The percentages of behavioral-emotional symptoms in the clinical range were alarmingly high in all LD groups in all scales, ranging from 15% to 59%. As about 7% would be expected in a normative sample with the cutoff criteria of 1.5 *SD* (93rd percentile), children with LD demonstrated about 2 to 8 times more clinical range problems than the normative Finnish sample. Especially high percentages were found in Affective, Anxiety, and ADHD problems, as they were reported in above 37% of the sample. Earlier research has reported varying percentages of behavioral-emotional problems among children with LD. For example, among those with RD, percentages of individuals with depression have varied from 10% (Willcutt et al., 2013) to 30% (Daniel et al., 2006), with anxiety from 12% (Carroll et al., 2005) to 23% (Goldston et al., 2007), and with conduct/oppositional or disruptive behavior from 8% (Willcutt et al., 2013) to 25% (Goldston et al., 2007). The percentages among children with MD have varied from 7% having depression (Willcutt et al., 2013) to 24% showing internalizing problems (Auerbach et al., 2008), and 20% having oppositional behavior (Willcutt et al., 2013) to 27% showing externalizing problems (Auerbach et al., 2008). Our percentages were somewhat higher. However, recently, Altay and Görker (2018) reported similarly high percentages of psychiatric disorder (82% ADHD, 46% phobia, and 26% oppositional defiant disorder) in their sample of 80 Turkish children attending psychiatric care and diagnosed with LD in reading, writing, or math.

There are several possible reasons for the high percentages in our sample. The sample consisted of children with clear LD who were referred to the CLD specialized in LD, and we used a strict criterion for LD (–1.5 *SD*). It can also be speculated that there was a referral bias toward children with co-occurring learning-related and psychological well-being problems. Although children with primary emotional problems are not referred to the CLD, it is plausible that parents and teachers are more concerned if they notice emotional distress in addition to LD. On the contrary, our sample is conservative in terms of behavioral-emotional problems, as children with primarily psychiatric problems are not referred to the CLD. In sum, our findings together with previous research indicate that children with LD have elevated risk for behavioral-emotional distress, especially Affective, Anxiety, and ADHD problems, and underscore the need for research on shared emotional, social, and neurobiological substrates underlining learning and behavioral-emotional difficulties.

Most of the behavioral-emotional symptoms rated to be in clinical range were reported only by teachers, especially Affective and ADHD problems (large effect size) as well as Anxiety and Oppositional Defiant problems (moderate effect size) were more prominent in school. Problems reported by both teacher and mother were few; however, ADHD problems were often manifested in both contexts. Although the discrepancy between the reports is in line with results on informant discrepancies (e.g., de los Reyes et al., 2013; van der Ende et al., 2012), it is in contrast with the findings suggesting that parents report more symptoms than teachers among children with LD (Dahle et al., 2011). Earlier, low informant agreement has been found, especially in internalizing problems (Salbach-Andrae et al., 2009; Stanger & Lewis, 1993; Youngstrom et al., 2000), but in our data, differences were detected also in externalizing problems. Our results suggest that there is contextual variation in the behavioral-emotional problems of children with LD, and the problems manifest more commonly in school than at home. This may signal that learning situations comprising challenging tasks and frequent demands (e.g., instructions, new assignments), expectation of failure, and comparison with better achieving peers are especially stressful for students with LD. It may also indicate that children with LD can identify the origin of their distress, and it does not necessarily generalize outside the learning context. Alternatively, our finding may be an indication that teachers are better informed about behavioral-emotional problems and are more skilled in recognizing them due to their experience with children (see Nelson & Harwood, 2011a), especially in Finland, where teachers have master’s degrees in education. However, it is also plausible that teachers have conflated learning-related difficulties with emotional ones, or there may have been uncontrollable factors (e.g., lack of familiarity with the child). The findings indicate that contextual variation needs to be further considered and underlie the importance of employing both parents and teachers as informants in future research and practice.

Although only a few differences emerged between LD types using the categorical approach, several findings raised concern about children with MD-only, and especially boys with MD-only. First, in all types of behavioral-emotional symptoms rated to be in the clinical range, the highest percentages were detected among children with MD-only (20%–56% among girls and 25%–59% among boys). Second, more boys with MD-only than expected were rated to have anxiety and ADHD, and among boys, more severe MD-only added anxiety symptoms. This is in line with the study by Graefen et al. (2015) showing more internalizing problems among boys than girls with MD-only. Third, severity of MD-only was also associated with more externalizing symptoms (i.e., ADHD, Oppositional Defiant, and Conduct problems) in both parental and teacher ratings, and additionally, with more internalizing symptoms (Affective and Somatic) in teacher ratings. This finding partly concords with Wu et al. (2014), who found that math achievement level was associated with externalizing, but not with internalizing symptoms using parent ratings. Earlier research has shown that math anxiety is common among children with MD (e.g., Auerbach et al., 2008), and our findings on internalizing symptoms may be seen as in line with this research. However, our analyses also indicate high percentages of externalizing problems among children with MD-only, and MD severity was associated with increased externalizing symptoms, which suggests that children with MD-only are at an elevated risk for increased emotional distress in addition to anxiety or math-specific anxiety. This should be taken into account in research on math anxiety. Awareness of the elevated risk for behavioral-emotional problems should be considered in math pedagogy and in preventive and supportive measures, as especially children with severe MD may need support for psychological well-being.

In the present data, RD-only was associated solely with an elevated percentage of Affective problems among girls, and RD severity added Somatic symptoms in boys. The comorbid group (RDMD) did not show more problems than the single deficit groups, which is in contrast with the study by Willcutt et al. (2013) in which the subgroup with comorbid RDMD showed more internalizing problems than the groups with single deficits. However, the percentages found in our data are more in line with those found in the study by Martínez and Semrud-Clikeman (2004), as they did not find differences between LD types. It might be that children with clear disabilities both in reading and math are more easily identified, and individual educational plans with adjusted academic goals are designed for them early on. Thus, they may be provided with more and earlier support than those with a single deficit, which may shelter them from psychological distress. Unfortunately, our data did not provide information about children’s own experiences or the support provided. Thus, future research should target the support provided and its effects on well-being, also taking the long run into account.

### Study Limitations

Some limitations typical of clinical data should be considered when interpreting our results. The participants were referred to the CLD due to learning problems. Therefore, children demonstrating *primarily* behavioral-emotional problems were not represented in the data. Thus, our findings on behavioral-emotional problems may even be conservative; that is, a higher incidence of problems would presumably have been found if children with known behavioral-emotional problems with comorbid LD were included. Our sample consisted of children with rather severe LD, as their learning difficulties were evident at school before they were referred by a psychologist to be further assessed at the CLD. It should also be noted that all the participants had either RD or MD based on their score being ≤−1.5 *SD*; however, we did not use a buffer zone and, therefore, a child with a score of −1.49 was designated as not having LD in that specific domain. However, using a buffer zone would not have altered the percentages of children with LD demonstrating behavioral-emotional problems or the differences between school and home.

Furthermore, although the service at the CLD is free, it is plausible that there are uncontrollable referral biases (e.g., children whose parents are supposed to be willing to go through the assessment process are referred or families with multiple psychosocial problems may fail to search for specialized help). These possible referral biases must be considered when generalizing the findings, even though their nature can only be speculated. Even though the participants were probably rather representative of the children with LD living in Central Finland, the results should be viewed with caution, as some of the data were collected 30 years ago, but removing the cohort assessed in 1985 to 1994 and having somewhat more behavioral-emotional problems did not change the results. The features of the Finnish school system should also be considered when generalizing the findings (e.g., inclusion of LD students in mainstream, no diagnosis is required for special educational support). Besides, the ASEBA norms used to define the cutoff for clinical range problems date to the beginning of the 21st century, and because it is not known, for instance, how children’s behavior, Finnish society, teachers’ or parents’ expectations or views for child behavior have changed, the percentages reported should be viewed as tentative. It should be remembered that the children with scores in the clinical range in behavioral-emotional symptoms are not equal to children with psychiatric diagnoses, as the percentages were based on a questionnaire and one informant only. As our data were not longitudinal, no causal inferences can be made, but there are indications that behavioral-emotional problems of children with LD tend to occur after school entrance (Parhiala et al., 2015).

### Implications

The finding indicating that a high percentage of children with LD demonstrated behavioral-emotional symptoms of clinical range, especially in the school context, underscores the importance of teachers’ awareness of behavioral-emotional problems among students with LD. It draws attention to the schools, to teachers and teacher education, and to the need for screening children with LD for behavioral-emotional symptoms. Similar to the meta-analysis conducted by Nelson and Harwood (2011a), our results suggest that teachers are valuable informants when behavioral-emotional problems are assessed, and they should be actively involved in the assessment along with parents. Thus, they should be provided with an up-to-date understanding of comorbidity of learning and well-being problems and on how to support psychological well-being. Accordingly, schools should have routines and strategies for identifying and providing support for students in need of it for both learning and psychological well-being.

In addition to individual targeted support, universal school-based promotion programs for well-being are needed. The results suggest that symptoms may manifest differently in different contexts, or that adults in these contexts are prone to observe or rate them differently due to their different perspectives. This underscores the need for multidisciplinary collaboration and incorporation of parents and teachers in both the assessment process and support provision. As differing ratings may contain even more information than if the informants agreed (van der Ende et al., 2012), relying on one informant or requiring agreement between the informants might lead to under-identification of children’s emotional distress. Therefore, the discrepant observations of the informants should be embraced as clinically relevant information, and the field should move toward theoretical conceptualizations of behavioral-emotional symptoms among children with LD explicitly incorporating contextual features (see Dirks et al., 2012) and search for further understanding on the origins of the differences between parents and teachers.

In a similar vein, clinicians working in child psychiatry with children experiencing psychiatric problems should assess the children’s academic history and consider comorbid LD. This requires adaptation of a holistic approach comprising assessment of cognitive, behavioral-emotional, and academic development and provision of support for both psychological well-being and academic skills. In future studies, LD-type and gender-related differences and severity of academic difficulty need to be considered, and specific symptom scales, instead of only internalizing and externalizing broad-band scales, should be included. More specifically, future research on the behavioral-emotional problems occurring especially in school among children with MD should aim to gain insight into the reasons and mechanisms behind the association. This understanding would be of utmost relevance for planning well-targeted interventions, which should consider the context and the person as a whole (i.e., his or her motivation, feelings, and skills, see for example, Ganley et al., 2021; Koponen et al., 2021).
